# Interstitial Lung Disease in Connective Tissue Disease: A Common Lesion With Heterogeneous Mechanisms and Treatment Considerations

**DOI:** 10.3389/fimmu.2021.684699

**Published:** 2021-06-07

**Authors:** Tihong Shao, Xiaodong Shi, Shanpeng Yang, Wei Zhang, Xiaohu Li, Jingwei Shu, Shehabaldin Alqalyoobi, Amir A. Zeki, Patrick S. Leung, Zongwen Shuai

**Affiliations:** ^1^ Department of Rheumatology and Immunology, The First Affiliated Hospital of Anhui Medical University, Hefei, China; ^2^ Division of Rheumatology/Allergy and Clinical Immunology, University of California, Davis, Davis, CA, United States; ^3^ Rheumatology, First Hospital of Jilin University, Changchun, China; ^4^ Department of Pharmacy, The First Affiliated Hospital of Anhui Medical University, Hefei, China; ^5^ Department of Pathology, The First Affiliated Hospital (Yijishan Hospital) of Wannan Medical College, Wuhu, China; ^6^ Department of Radiology, The First Affiliated Hospital of Anhui Medical University, Hefei, China; ^7^ Internal Medicine - Pulmonary, Critical Care, and Sleep Medicine, Brody School of Medicine, Greenville, NC, United States; ^8^ University of California (U.C.), Davis, Lung Center, Division of Pulmonary, Critical Care, and Sleep Medicine, Department of Internal Medicine, U.C. Davis School of Medicine, University of California, Davis, Davis, CA, United States

**Keywords:** connective tissue disease, interstitial lung disease, genetics, environmental exposure, autoantibodies, signs and symptoms, risk assessment, therapeutics

## Abstract

Connective tissue disease (CTD) related interstitial lung disease (CTD-ILD) is one of the leading causes of morbidity and mortality of CTD. Clinically, CTD-ILD is highly heterogenous and involves rheumatic immunity and multiple manifestations of respiratory complications affecting the airways, vessels, lung parenchyma, pleura, and respiratory muscles. The major pathological features of CTD are chronic inflammation of blood vessels and connective tissues, which can affect any organ leading to multi-system damage. The human lung is particularly vulnerable to such damage because anatomically it is abundant with collagen and blood vessels. The complex etiology of CTD-ILD includes genetic risks, epigenetic changes, and dysregulated immunity, which interact leading to disease under various ill-defined environmental triggers. CTD-ILD exhibits a broad spectra of clinical manifestations: from asymptomatic to severe dyspnea; from single-organ respiratory system involvement to multi-organ involvement. The disease course is also featured by remissions and relapses. It can range from stability or slow progression over several years to rapid deterioration. It can also present clinically as highly progressive from the initial onset of disease. Currently, the diagnosis of CTD-ILD is primarily based on distinct pathology subtype(s), imaging, as well as related CTD and autoantibodies profiles. Meticulous comprehensive clinical and laboratory assessment to improve the diagnostic process and management strategies are much needed. In this review, we focus on examining the pathogenesis of CTD-ILD with respect to genetics, environmental factors, and immunological factors. We also discuss the current state of knowledge and elaborate on the clinical characteristics of CTD-ILD, distinct pathohistological subtypes, imaging features, and related autoantibodies. Furthermore, we comment on the identification of high-risk patients and address how to stratify patients for precision medicine management approaches.

## Introduction

Connective tissue disease (CTD) is a heterogeneous group of inflammatory disorders that can affect bone, cartilage, tendons, ligaments, muscle, joints, blood vessels, and even specific organs. Many CTDs such as systemic lupus erythematosus (SLE), rheumatoid arthritis (RA), Sjogren’s syndrome (SS), polymyositis (PM)/dermatomyositis (DM), systemic sclerosis (SSc), and mixed connective tissue disease (MCTD) are autoimmune mediated. The major pathological features of autoimmune mediated CTD are chronic inflammation of blood vessels and connective tissues, which can affect any organ leading to multi-system damage.

The human lung is particularly vulnerable to such damage because anatomically it is abundant with collagen and blood vessels that are essential for metabolic, endocrine, and immune functions. Various components of the respiratory system including the airways, vessels, parenchyma, pleura, and respiratory muscles may also be involved (1). In such cases, this manifests clinically as pulmonary interstitial diseases, pulmonary vascular diseases, diffuse alveolar hemorrhage, bronchiolitis, pulmonary parenchymal nodules, pleural lesions or effusions, respiratory muscle weakness, and aspiration pneumonia. Understanding the diverse clinical manifestations and high mortality of interstitial lung disease (ILD) in patients with CTD is important and highly relevant to the practice of rheumatology.

The heterogeneity in disease severity, underlying mechanisms, and clinical manifestations of CTD-ILD can be perplexing. Clinical and research-based rheumatologists are faced with several challenges in the diagnosis and management of CTD-ILD: (a) despite extensive effort, the precise mechanisms that drive CTD-ILD remain unclear; (b) it is easy to miss or misdiagnose patients when they present with pulmonary involvement but without clear immunological manifestations; (c) there is no standard protocol for evaluating a given patient’s condition and assessing disease progression, e.g. when we confirm that the patient is deteriorating, there are no effective methods or biomarkers to determine whether the patient’s deterioration is due to the progression of ILD or other reasons, such as infection or drug-induced causes; (d) CTD-ILD is difficult to treat. CTD-ILD has a more favorable prognosis than idiopathic interstitial pneumonia (IIP) because it can be treated with glucocorticoid (GC) and immunosuppressive agents (2, 3). However, the side effects of these medications, treatment dose(s), and clinical course can vary greatly between patients. Further, once the patient progresses to pulmonary fibrosis, the prognosis becomes less optimistic. Owing to the complexity of treatment, tailoring treatment protocols for CTD-ILD requires vigorous effort and a multidisciplinary team approach often including close collaboration with the patient’s pulmonologist (4, 5).

## Pathogenesis of CTD-ILD

**Figure 1:**
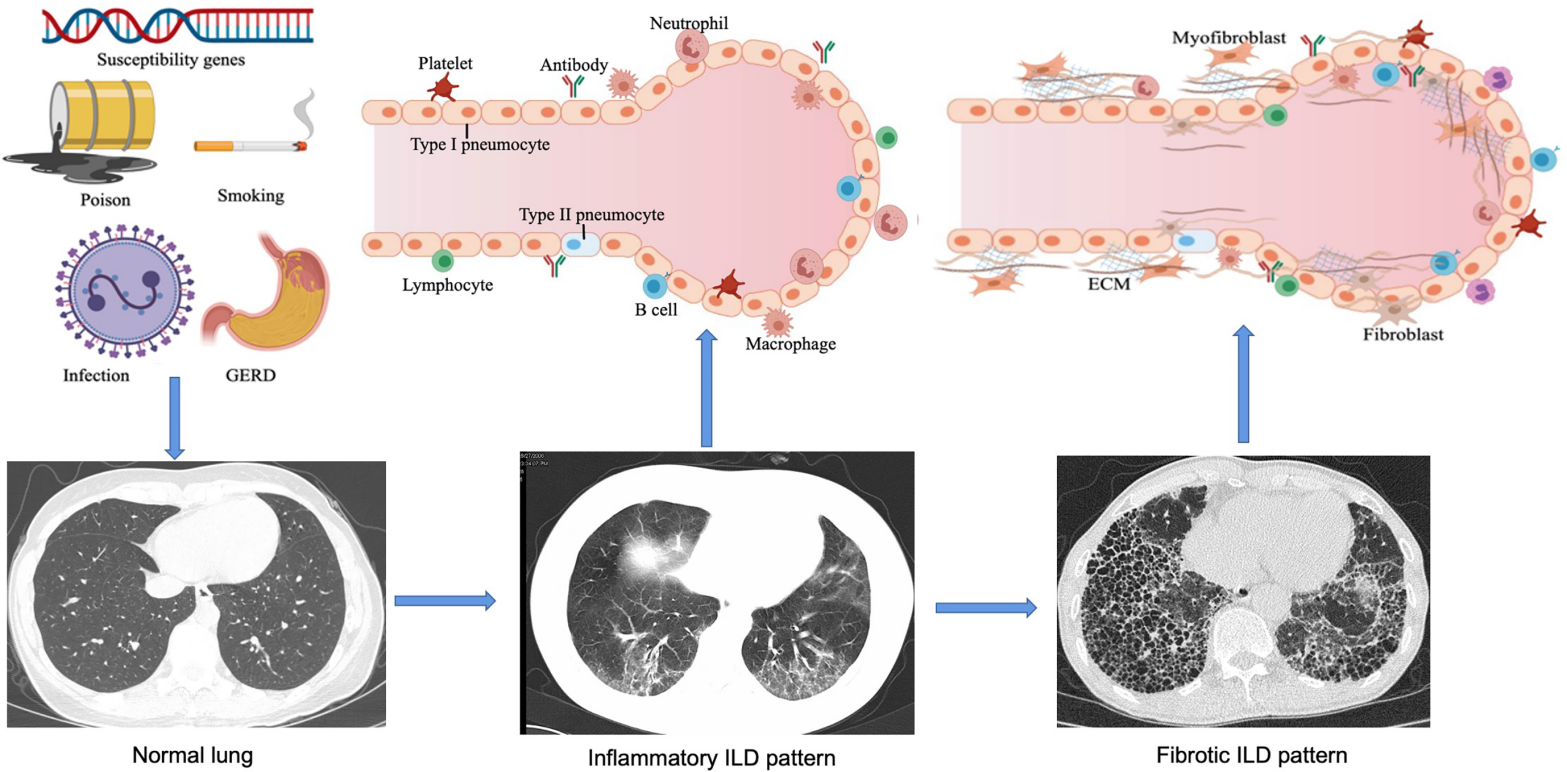
Pathogenesis and development of ILD. In genetically susceptible individuals, external factors such as smoking, environmental chemicals, infections and gastroesophageal reflux disease (GERD) can lead to epithelial cell injury and aberrant repair, alveolar macrophage activation, neutrophil recruitment, and oxidative stress. Over time, increased ECM turnover will result in the development of fibrosis. With these exposures the host's immune tolerance is broken leading to chronic inflammation from cellular and humoral autoimmunity, endothelial cell dysfunction, granuloma formation, and alveolar macrophage activation thus further aggravating inflammation. As the disease progresses, interstitial pneumonia changes from an early alveolitis (increased alveolar space content of inflammatory products, reflecting inflammatory ILD pattern) to a transition period (thickened alveolar septum and the deposition of collagen fibers, significantly reduced capillary beds), and eventually ending in alveolar structural destruction and fibrosis. This figure is created with MedPeer.

ILD refers to a group of heterogeneous non-neoplastic diseases belonging to the category of diffuse parenchymal lung diseases (DPLDs) that affect alveolar epithelial cells, pulmonary capillary endothelial cells, basement membrane, perivascular, and lymphoid tissues. CTD-related ILD (CTD-ILD) can be similar to the IIPs [e.g. idiopathic pulmonary fibrosis (IPF)], especially when the lung is the only organ involved, or the lung injury happened in connective tissues prior to the extrapulmonary manifestations. Genetics (6), environment (7, 8), and immunological factors (9, 10) could be involved in the pathogenesis of CTD-ILD (**Figure 1**). Here, we discuss our current understanding of genetic predisposition, the environment, and immune regulation of CTD-ILD.

### Genetics

The application of high throughput genetic analysis has led to the identification of a number of genetic loci that are associated with the risk of developing CTD-ILD and worse prognosis (11–13). We will discuss major findings regarding the effects of genetic susceptibility and its association with the poor prognosis seen in CTD- ILD. Rare pathogenic mutations in telomere maintenance genes and chromosome-protected terminal telomere shortening are related to pulmonary fibrosis. Newton et al. have examined telomere-related variants in patients with ILD (14). Patients with rare telomere-related variants *TERT, TERC, PARN*, or *RTEL1* exhibit various forms of pulmonary fibrosis, ranging from IPF, interstitial pneumonia with autoimmune features (IPAF), to CTD-ILD. Interestingly, there exists statistical correlations in the mean diagnostic age of patients with different gene mutations. TERC mutation carriers were diagnosed at the earliest age (51 years old) and had a higher incidence of hematological comorbidities. The age of diagnosis was highest in *PARN*, followed by *RTEL1, TERT* and *TERC*, and is consistent with the order of average telomere length (14). Studies have reported that shorter leukocyte telomere length is associated with faster decline in lung function and shorter duration of transplant-free survival in patients with IPAF and CTD-ILD (11–13).

The strongest risk factor for the development of IPF has been identified as the gain-of-function of the *MUC5B* promoter variant *rs3570595*0, which is observed in more than two-thirds of patients with IPF and accounts for 30% to 35% of the risk of developing disease (15–23). Several studies have demonstrated that *MUC5B* minor alleles correlate with the deterioration of lung function and survival rate of IPAF and CTD-ILD (11–13). Compared with non-CTD-ILD controls, the frequency of *MUC5B* minor allele frequency is higher in CTD-ILD, especially the RA-ILD subgroup (24). Notably, Juge et al. examined the effects of *MUC5B* promoter variant *RS35705950* on RA-ILD and showed that the *MUC5B* promoter variant was associated with RA-ILD, with a characteristic interstitial pneumonia imaging pattern (6). Genetically driven *MUC5B* overexpression of *MUC5B* protein can hinder cilia clearance or disrupt normal lung repair mechanisms (25). Collectively, these studies support that *MUC5B* is involved in the pathogenesis of CTD-ILD and may be a therapeutic target.

Similar to familial IPF patients, data from exome-sequencing revealed the presence of *TTR*, *RTL1, PARN*, or *SFTPC* mutations in RA-ILD patients, indicating the contribution of IPF-linked genes in RA-ILD susceptibility (26). In addition to common genetic features, RA-ILD and IPF patients have overlapping clinical features, such as older age, and higher occurrence in males and in cigarette smokers (27, 28). Cumulative evidence has also indicated that a number of genetic loci are associated with susceptibility to SSc-ILD, including *CTGF* (also known as *CCN2*, encoding connective tissue growth factor), *CD247* and *IRF5* (29–32). Fingerlin et al. reported that two *HLA* alleles in the high linkage disequilibrium are associated with pulmonary fibrosis (DRB1 * 15:01 and DQB1 * 06:02) (33, 34). These susceptibility genes are similar to the previous ILD-related loci associated with PM/DM (34–36). A list of CTD-ILD susceptibility genes currently identified in the literature is shown in the **Table 1**.

**Table 1:** Susceptibility genes in CTD-ILD.

Disease	Susceptibility genes
RA-ILD	DRB1*16:02, DRB1*15:02 (37–39)
	*TERT、RTEL1、PARN or SFTPC* (26)
	*MUC5B* (6)
SSc-ILD	HLA-B*62, HLA-C*06, DRB1*11 (40–42)
	DPB1*03:01, DR51 (43, 44)
	*CD226, MMP12, SFTPB, CTGF, HGF, IRAK1, TCRBV, IRF5* (29, 45–51)
	*CD247 (* 30, 31)
PM/DM-ILD	DRB1*03, DRB1*01:01, DRB1*04:05 (35, 36)
	DQB1*06:02 (34)
CTD-ILD	*TERC、TERT* (52)

TERT, telomerase reverse transcriptase; RTEL1, telomere-elongation helicase-1; PARN, polyadenylation-specific ribonuclease deadenylation nuclease; SFTPC, surfactant protein C; MUC5B, recombinant Mucin 5 Subtype B; HLA, Human Leukocyte Antigen; CD, clusters of differentiation; MMP, matrix metalloproteinase; SFTPB, surfactant protein B; CTGF, connective-tissue growth factor; HGF, hepatocyte growth factor ; IRAK, IL-1 receptor-associated kinase; TCRBV, T-cell receptor-**β** variable; IRF5, recombinant interferon regulatory factor 5; TERC, telomerase RNA component; TERT, telomerase reverse transcriptase.

There are epigenetic mechanisms including DNA methylation, post-translational histone modification, and non-coding RNA in IPF. The differences in their DNA methylation pattern may influence the expressions of many target genes and microRNAs (miRNAs), as well as the regulatory sites of genes involved in IPF (53, 54). Based on comparative analysis of genome-wide DNA methylation together with gene expression patterns in lung tissues from IPF patients and normal controls, Sanders et al. demonstrated that *ZNF467* and *CLDN5* with hypermethylation are down-regulated, whereas *TP53INP1* and *DDAH1* with hypomethylation are up-regulated in IPF (55). Studies on histone modifications mainly revealed the involvement of epithelial-mesenchymal transition (EMT), apoptosis, and the prostaglandin E2 pathway (56). Histone deacetylase inhibitors can eliminate the differentiation of fibroblast-myofibroblasts induced by transforming growth factor-β1 (TGF-β1), restore the expression of surfactant protein-C in alveolar epithelial type II cells, and mitigate bleomycin-induced pulmonary fibrosis (57, 58). Histone deacetylase inhibition can also increase *Fas* expression, which exhibited low level expression in fibroblasts from both IPF patients and mice with experimental pulmonary fibrosis, and restore sensitivity to *Fas*-mediated apoptosis, indicating the key role of histone modification in the development of anti-apoptotic fibroblasts (59). Changes in miRNA profiles have been observed in IPF patients and mouse models of fibrosis, including the down-regulation of some microRNAs, such as *let-7*, *mir-29* and *mir-30*, members of the *miR-200* family, and upregulation of miRNAs, such as *mir-155* and *mir-21*. Regulating the expression of miRNAs can attenuate or aggravate IPF, which exploits a new era for a miRNA-mediated therapeutic approach to the treatment of IPF (60–64). There is increasing evidence to support the involvement of epigenetics in the pathogenesis of IPF, however, there are limited studies on the correlation between epigenetics and CTD-ILD. Therefore, relevant studies are needed to address this relationship.

### Environmental Factors

Multiple environmental factors including gastroesophageal reflux disease (GERD), infections (7, 65), environmental chemicals, toxic substances, drugs (66–68), and tobacco smoke are associated with inflammatory lung injury (8, 69, 70). The prevalence of GERD in ILD can be as high as 94% (71–73). Animal studies have shown that chronic aspiration leads to pulmonary fibrosis (74). It is postulated that GERD-associated chronic micro-aspiration induces repetitive lung injury, resulting in pneumonitis, increased epithelial permeability, fibrotic hyperplasia, and ultimately pulmonary fibrosis (75). A murine model of aspiration-induced lung injury model exhibited extensive collagen deposition by the second week (76), and revealed reflux containing bile acids, elevated TGF-β levels, and prominent fibroblast proliferation (77). On the contrary, meta-regression analysis adjusted for smoking suggested chronic micro-aspiration in GERD is not associated with IPF (78). In addition to the contribution from acidic stomach contents, *Helicobacter pylori* in gastric juice can also cause lung injury, and thereby, promote progressive pulmonary fibrosis (79, 80).

Although Epstein-Barr virus is a prime suspect, other viruses and bacteria (e.g., retroviruses, parvoviruses, mycobacteria, Mycoplasma species, and Borrelia species) have also been implicated in inflammatory lung injury (7).

Particulate matter and toxic chemicals in tobacco smoke can activate immune cells, recruit inflammatory cells, and lead to the influx of various immune cells into the lungs, and this in concert can eventually lead to ILD (81, 82). However, the effect of smoking on CTD-ILD is unclear. Among various autoimmune diseases, RA is most definitely associated with smoking. Epidemiological studies have demonstrated that people exposed to tobacco smoke are at a higher risk of developing seropositive RA, and in predisposed individuals, smoking can promote the production of anti-cyclic citrullinated peptide (anti-CCP) antibodies (83–85). Notably, increased prevalence of emphysema and decreased survival have been noted in patients with SSc who smoke heavily, indicating the adverse effects of smoking in SSc (86–88).

More than 600 drugs have been reported to cause severe pulmonary injury (See pneumotox.com for a list of drugs that have been reported to cause lung toxicity). Multiple drugs used in treating cardiovascular diseases, anti-inflammatory, antimicrobial, and cancer immunotherapies as well nonbiologic and biologic disease-modifying anti-rheumatic drugs (DMARDs) have also been associated with severe lung injury (89). Anti-rheumatic drug-induced ILD (DILD) is not uncommon and can be driven *via* dose-dependent toxicity and immune-mediated allergic reaction (90). Risk factors of DILD include genetic susceptibility (91), age, sex, smoking, underlying lung disease such as pre-existing ILD, bronchiectasis, chronic obstructive pulmonary disease, dosage of drugs, and interactions with concomitant drugs and previous treatment, such as chest radiotherapy (68, 92, 93). Clinically, it is difficult to distinguish DLID from other interstitial pneumonias. Multiple imaging patterns can result from the same drug, and vice versa (68). Similarly, it is challenging to diagnose DILD due to presenting signs and symptoms that are often very similar to other ILDs.

The diagnosis of DILD is based on the following: (a) an exposure to the causative agent and presenting concomitant respiratory signs and symptoms which are consistent with previous reports, (b) ruling out other causes of lung damage including infection, cancerous lymphangitis, radiotherapy-induced pneumonitis, congestive heart failure, and exacerbation of pre-existing ILD, (c) alleviation of symptoms after discontinuation of the offending drug and relapse after reapplication. Rheumatologists may face several challenging clinical scenarios including the development of initial symptoms after drug withdrawal, or continued aggravation of clinical symptoms despite drug discontinuation. In addition, when a patient develops ILD during the treatment of a rheumatologic disorder, it is difficult to determine whether it is drug-induced or whether it is complicated by CTD. Similarly, when CTD-ILD patients progress or worsen during treatment, it is difficult to determine whether this is due to a drug side effect or the natural progression of disease.

Importantly, clinicians need to be aware of what medications are associated with DILD. The main nonbiological DMARDs include gold (94, 95), penicillamine (96), sulfasalazine (97), tacrolimus  (98, 99), methotrexate (MTX) (93, 100–102), and leflunomide (103–105), and the biological agents mainly include anti-TNF, anti-CD20, and cytokine monoclonal antibodies (106, 107). Although MTX-induced ILD is well-established by many studies, there is some emerging conflicting evidence suggesting no association between MTX and RA-ILD (108). For example, a study of MTX use and the risk of ILD in RA patients demonstrated that there was no further increase in risk associated with MTX treatment (109). Other studies have also reported similar findings suggesting that MTX may delay the onset of ILD (110, 111). However, the overall picture and recommendation indicates that exposure to any of the aforementioned drugs could potentially lead to DILD. Therefore, the alert clinician must be aware of this possibility because early recognition could lead to the earlier initiation of therapy.

### Immunological Factors

Both innate and adaptive immune system are potential culprits for the pathogenesis of CTD-ILD. For example, B cells contribute to autoimmune ILD (112) with studies showing the presence of extensive B cell infiltrations in lung tissue samples of SSc-ILD patients (9). Compared with IIP, RA-ILD is distinguished by its prominent increase in CD4^+^ cells and follicular B cell hyperplasia in the lung (113, 114). In patients with RA-ILD and SSc-ILD, T cells release fibrogenic mediators which subsequently stimulate fibroblasts and prime the fibrotic response (115). In SSc-ILD, alveolar macrophages become M2 polarized upon induction by the Th2 cytokines IL-4 and IL-10, suggesting that the M2/Th2 pathway is involved in the pathogenesis and development of SSc-ILD (116). Autoantibodies are also associated with CTD-ILD, with some antibodies occasionally related to the course and severity of the disease, reinforcing the notion that humoral immunity is involved in the pathogenesis of CTD-ILD (117, 118). This is discussed in greater detail in the CTD-ILD related autoantibody section below.

Toll-like receptors (TLRs), key components of innate immunity, have multi-faceted effects on ILD in patients with CTD. TLRs have been proposed as markers of ILD progression (10). Correlation studies showed that TLR2 (119) and TLR9 (120) are profibrotic while TLR3 (121) is anti-fibrotic in pulmonary fibrosis. On the other hand, TLR4 can be either profibrotic (122) or anti-fibrotic (123) depending on the micro-environment. TLR2, TLR3 mRNA in bronchoalveolar lavage fluid (BALF) T-lymphocytes and peripheral blood monocytes, are over-expressed in CTD-ILD compared with healthy controls, suggesting that TLRs may be involved in the pathogenesis of CTD-ILD (124–126). The contribution of other innate players on CTD-ILD remains to be explored. Understanding the mechanistic roles of immune cell activities in CTD-ILD will help in the development of innovative and novel therapeutic approaches.

In genetically predisposed individuals, the pathogenesis of CTD-ILD involves recurrent alveolar injury and dysfunctional healing which are key causative mechanisms in the development of pulmonary fibrosis. Pulmonary fibroblasts are activated to produce extracellular matrix as inflammatory cells enter and infiltrate the lung interstitial and alveolar spaces. This leads to an imbalance of collagen formation and degradation, resulting in collagen over-accumulation in the lung (127). Epithelial and mesenchymal cells, as well as components of the innate and adaptive immune system, lead to a favorable microenvironment that promotes disease pathogenesis (128). These factors together, contribute to the chronic inflammation, gradual destruction of functional lung parenchyma, replacement by collagen, thus, ultimately leading to pulmonary fibrosis, respiratory failure, and early mortality.

## Clinical Characteristics of CTD-ILD

Clinical manifestations of CTD-ILD include constitutional and respiratory symptoms, but these are rather non-specific. The common constitutional symptoms include fatigue, fever, and weight loss. The most common respiratory symptoms include exertional dyspnea, exercise intolerance, and dry (or non-productive) cough with slow progression over the time. Other concomitant symptoms may include chest pain, palpitations, tachypnea, and hemoptysis. In addition to pulmonary involvement, CT-ILD can also involve the mucocutaneous, musculoskeletal, neurological, gastrointestinal, cardiac, and hematologic systems.

Generally speaking, compared with IIP, CTD-ILD patients are more likely to be younger, female, and non-smokers. However, the exact frequency of CTD-ILD is not known. Although the incidence and prevalence vary between studies, it is estimated that 10 to 90% of patients with CTD will have evidence of pulmonary involvement during their lifetime. The types of pulmonary manifestations may vary by underlying CTD diagnosis (**Table 2**). The prevalence and mortality of ILD for each CTD are different, and the prevalence of ILD secondary to various CTDs varies as follows: 1 to 15% in SLE (106), 6.5 to 33% in RA (107, 108), 19.9 to 86% in PM/DM (109, 110), 86% in anti-Jo-1 positive patients (111), 40 to 91% in SSc (112, 113), 47 to 90% in MCTD based on radiologic feature (108, 114), and 9 to 20% in SS (115). Related reports indicate the mortality of ILD is 20% in RA-ILD (116, 117), 12 to 44% in PM/DM (118), and a 10-year mortality of up to 40% in SSc (119). In patients with RA and SSc, the 5-year mortality is 3-fold higher than that without ILD (120, 121).

**Table 2:** Characteristics of lung involvement in different CTD-ILD.

Manifestation	RA	SSc	SS	SLE	PM/DM	MCTD
Airways disease	++	–	++	+	–	+
ILD	++	+++	++	+	+++	++
*NSIP*	++	+++	++	++	+++	++
*UIP*	+++	+	+	+	+	+
*OP*	++	+	+	+	+++	+
*DAD／AIP*	+	+	+	++	++	+
*LIP*	+	–	++	+	–	–
DAH	+	+		++	+	+
Pleural disease	++	–	+	+++	–	+
Vascular disease	+	+++	+	+	+	++
Pulmonary hypertension	+	+++	+	+	+	+
Parenchymal nodules	+	–	–	–	–	–
Respiratory muscle disease	–	–	–	+	++	+
Aspiration pneumonia	–	+++	–	–	+	+

NSIP, nonspecific interstitial pneumonia; UIP, usual interstitial pneumonia; OP, organizing pneumonia; DAD, diffuse alveolar damage; AIP, acute interstitial pneumonia; LIP, lymphoid interstitial pneumonia; DAH, diffuse alveolar hemorrhage;

Prevalence of each manifestation is expressed as:

–, no prevalence; +, low prevalence; ++, medium prevalence; +++, high prevalence.

## Histological Classification of CTD-ILD

Histologically, CTD-ILD can be divided into 7 types including: usual interstitial pneumonia (UIP), nonspecific interstitial pneumonia (NSIP), desquamative interstitial pneumonia (DIP), respiratory bronchiolitis (RB), organizing pneumonia (OP), diffuse alveolar damage (DAD), and lymphoid interstitial pneumonia (LIP) (129, 130). These histological classifications provide a more comprehensive diagnosis of CTD presenting with ILD. We note that NSIP is the most common histopathologic type in CTD-ILD (with the exception of RA), however, UIP is the most common in IIP (131). In addition, the frequency and severity of fibroblastic lesions in CTD-ILD is lower than IPF-UIP (132). We also note that the coexistence of UIP and NSIP patterns is one of the most significant features that distinguishes CTD-UIP from IPF-UIP (133). The frequency of ILD pathological subtype from different underlying CTDs is shown in **Table 2** (134–137). Although the pathological classification of CTD-ILD is identical to that of IIP, some histopathologic features such as extensive plasma cell infiltration, increased lymphoid aggregates, and more germinal centers are considered to be characteristic histologic features of CTD-ILD as compared with IIP (138–140). Indeed, the clinical characteristics, therapeutic response to GC and immunosuppressive agents, and prognosis of CTD-ILD vary according to the pathological subtypes (**Table 3**).

**Table 3:** Clinical Characteristics, Response to Therapy and Prognosis of CTD-ILD Subtypes.

Pathological Subtype	Course	Clinical Manifestations	Imaging Findings	Pathologic Features	Therapeutic Effects to GC and IMS	Prognosis
AIP (141)	Acute: days to weeks	Fever, cough and progressive severe tachypnea	Bilateral ground-glass opacities and/or airspace consolidation	Diffuse alveolar damage	Yes	75% mortality in 6 months
OP (142–144)	Acute/ subacute: days to months	Fever, cough and dyspnea	Bilateral patchy peripherally located consolidations or ground glass opacities.	An excessive proliferation of fibrous tissue within the alveolar sacs and alveolar ducts	Yes	Spontaneous remissions are seen in about 50% of mild cases. Patients demonstrate a rapid symptomatic response to treatment and up to 80% achieve complete cure
UIP (145–147)	Chronic: months to years	Slowly progressive dyspnea and nonproductive cough	Honeycombing with a peripheral predominance	Patchy dense fibrosis causing remodeling of lung architecture	No	5- and 10-year survival are 43% and 15%, respectively, median survivals from the time of diagnosis is about 3 years
NSIP (147, 148)	Chronic: months to years	An insidious onset of shortness of breath over several months, accompanied by a cough	Bilateral ground-glass opacities in a basal and peripheral distribution	A temporally homogeneous inflammatory and fibrosing interstitial process	No	86%-92% 5-year survival and 26%-40% 10-year survival rates
LIP (149, 150)	Chronic: months to years	Progressive dyspnea and dry cough	Thickened bronchovascular bundles, nodules of varying sizes, and ground-glass opacities	Diffuse interstitial lymphocytic infiltrates with widened interlobular and alveolar septae	Yes	5-year mortality is 33% to 50% for all types of LIP despite treatment, with reported median survival times ranging from 5 years to 11.5 years

GC, glucocorticoid; IMS, immunosuppressive agents.

## Imaging Features of CTD-ILD

**Figure 2:**
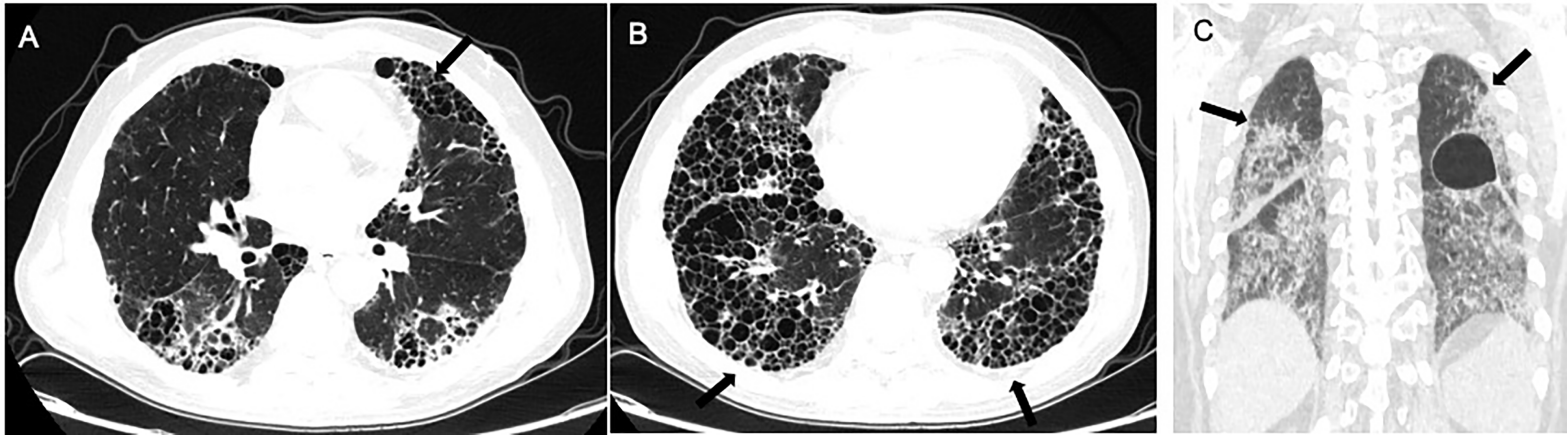
Radiological imaging pattern of CTD-ILD. **(A)** “anterior upper lobe” sign. **(B)** “exuberant honeycombing” sign. **(C)** “straight-edge” sign. All these signs are indicated by arrows.

Radiologically, high resolution computed topography (HRCT) scans can be effectively used to diagnose and identify disease and assess disease improvement or progression. CTD-ILD may manifest as a focal or a diffuse pulmonary abnormality, especially at the periphery of the lung, such as reticulation, ground-glass opacities (GGOs) (which refers to focal or diffuse veil-like opacification of the lung), and nodules. Imaging findings of the different kinds of pulmonary lesions vary with specific diseases and histopathologic patterns observed (151, 152). In addition to their respective characteristic imaging manifestations, certain radiological clues support the diagnosis of CTD-ILD (**Table 3** and **Figure 2**). For example, (a) In the combined NSIP-OP pattern (130, 153), CTDs such as idiopathic inflammatory myopathies (IIMs) or anti-synthetase syndrome (ASS) should be suspected when the fibrosis at the lung bases overlaps with an OP pattern. (b) In the combined DAD-IIP pattern, when the DAD is superimposed on another IIP pattern it may indicate the presence of CTD. Sometimes ASS manifests as acute respiratory failure with DAD superimposed on underlying IIP, however, this is not specific to ASS (151, 154). Atypical interstitial pneumonia may be due to unclassifiable or mixed imaging findings (155).

Chung et al. compared the CT manifestations of CTD-UIP and IPF-UIP, and found that there are three imaging manifestations with high specificity but low sensitivity for CTD-ILD, including (i) the “anterior upper lobe” sign and concomitant lower lobe involvement, (ii) “exuberant honeycombing” sign constituting greater than 70% of fibrotic portions of the lung, and (iii) “straight-edge” sign indicating lung basal fibrosis with sharp demarcation in the craniocaudal plane (155).

Finally, some extrapulmonary signs on HRCT support the diagnosis of CTD-ILD, such as esophageal or pericardial abnormalities, features of pulmonary arterial hypertension, evidence of airway disease, findings suggestive of bone and joint involvement, and soft-tissue calcifications (156).

## Clinical Significane of CTD-ILD Related Autoantibodies

There are multiple autoantibodies in the sera of patients with CTD, many of which are associated with interstitial lung injury (**Table 4**). Among the SSc-ILD-related antibodies, anti-topoisomerase antibodies are more likely to be associated with pulmonary fibrosis, while anti-RNA polymerase III antibodies are less likely to be associated with pulmonary fibrosis (157). Other antibodies have also been linked to increased lung fibrosis risk in SSc, including anti-U11/U12 ribonucleoprotein (RNP) antibodies, or anti-Th/To-RNP antibodies (158). Anti-U11/U12 RNP may be related to the severity of ILD (159–162). The correlation between anti-Scl-70 and the severity of ILD is unclear, however, anti-Ro52 antibody is associated with ILD and poor prognosis in SSc. The ANA of nucleolar pattern is also associated with pulmonary fibrosis in patients with SSc whereas anticentromere antibodies (ACA) are not. ANA patterns can be used to predict the risk of pulmonary fibrosis in patients with SSc (163).

**Table 4:** Autoantibodies and serological immune markers associated with CTD-ILD.

	PM/DM	SSc	RA	SS	MCTD
Autoantibodies and serological immune markers	MSAs	anti-Scl-70	RF	Anti-SSA/Ro	Anti-U1RNP
	anti-Jo-1	anti-U3RNP	Anti-CCP	anti-SSB/La	CIC
	anti- PL-12	anti-U11/U12RNP			C3
	anti- PL-7	anti-RuvBL1/2			CH50
	anti- KS	anti-EIF2B			
	anti- OJ	anti-PM-Scl			
	anti- EJ	anti-U1RNP			
	anti-Zo	anti-cardiolipin			
	anti-Ku	anti-Th/To			
	anti-MDA5	anti-Ro52			
	MAAs	anti-NOR90			
	anti-Ro52/60	nucleolar ANA			
	anti-U1RNP	ANCA			

MSAs, myositis-specific autoantibodies; MDA5, melanoma differentiation-associated gene 5; MAAs, myositis-associated antibodies; ANCA, anti-neutrophil cytoplasmic antibodies; RF, rheumatoid factor; Anti-CCP, anti-citrullinated peptide antibodies; CIC, circulation immunity compound.

In patients with autoinflammatory myopathy, myositis-specific autoantibodies (MSAs) and myositis-associated antibodies (MAAs) are associated with IIM-ILD, and those with anti-Ku antibodies have a higher risk of lung involvement (161). Single factor Cox hazards analysis showed that the presence of anti-aminoacyl-transfer RNA synthetase (ARS) antibodies indicates a better prognosis, and the presence of anti-synthetase antibodies might be used as a prognostic marker for PM/DM ILD patients. Sabbagh et al. discovered that patients with adolescent myositis with anti-Ro52 were more likely to develop ILD, have more severe disease, and have a worse prognosis (164). A related study demonstrated that anti-Ro52 antibodies and anti-Jo1 antibodies are usually present together (165), and anti-Ro52 antibody titers correlate with ILD severity (166). Compared to anti-Jo1 autoantibodies alone, adult patients with both autoantibodies were more prone to severe ILD, poorer response to various immunosuppressive drugs, and lower survival rates (167–169). Specifically targeted to scaffold attachment factor B (SAFB), anti-SAFB antibodies were detected in a small number of patients with SSc and/or PM/DM, and ILD. Anti-SAFB antibodies may be a novel CTD-related autoantibody associated with ILD (170).

Elevation of RF and anti-CCP antibodies in the serum and BALF are considered risk factors for RA-ILD (171–174). While anti-SSA/Ro and anti-SSB/La are associated with SS-ILD, it is interesting that the specificity of anti-La antibodies in lung involvement is higher than that of anti-Ro antibodies. Anti-U1-RNP, immune complex, complement C3 factor, and CH50 are highly expressed in MCTD-ILD. Anti-Ro52 antibodies are associated with MCTD-ILD (175). Anti-endothelial cell antibodies (AECAs) are associated with a high incidence of pulmonary fibrosis and severe diffusion abnormalities (176). In addition to these autoantibodies, other ILD-associated CTD autoantibodies and serological immune markers are listed in **Table 4** (177).

## Identifying High-Risk Patients

Rheumatologists should be vigilant about CTD-ILD, including the timely identification of high-risk patients. ILD is characterized by non-productive cough, fever, a gradual onset of exertional dyspnea, and fine bibasilar inspiratory crackles (“velcro” crackles). These signs and symptoms are non-specific and can be seen in a variety of pulmonary and/or heart diseases, and approximately 5% of patients have no symptoms when ILD is serendipitously diagnosed. One important consideration involves patients with negative autoantibodies and no extrapulmonary immune features who are eventually diagnosed with CTD-ILD after long-term follow-up. Since diagnosis of these patients can be challenging and delayed, regular assessments are required during clinic follow-ups to make the correct diagnosis. In a study on 1,044 Chinese CTD-ILD patients, 43.8% of them had a negative autoantibody serological test at the time of initial admission, however 25.1% seroconversions and 18.7% persistent negatives were found on subsequent follow-ups. In the latter group, most of these patients were finally diagnosed with CTD-ILD because of their emerging extrapulmonary features and/or need for lung biopsy (178).

Pulmonary dysfunction mainly manifests as a restrictive pattern with a decrease in total lung capacity (TLC), forced vital capacity (FVC), residual volume (RV), functional residual capacity (FRC), and diffusion capacity of carbon monoxide (DLCO). These pulmonary function test (PFT) findings reflect a restrictive ventilator defect due to pulmonary interstitial fibrous tissue hyperplasia leading to increased diffusion distance, and decreased diffusion capacity.

An emerging phenotype called “progressive fibrosing-ILD” (PF-ILD) that is characterized by significant decline in FVC (relative decline of ≥5-10%) and DLCO (relative decline of ≥5-15%) over a period of time ranging between 6 to 24 months, is associated with increased mortality (179, 180). Other criteria included worsening symptoms and increased fibrotic changes on HRCT (181). CTD-ILD can present with a PF-ILD phenotype if they meet the above criteria (179, 181). A recent decline in FVC and DLCO are independent predictors of decreased survival rate in SSc-ILD (157, 182–184).

On thoracic imaging, loss of lung volume, parenchymal reticulations, and GGOs are common. Typical pulmonary HRCT findings in patients with CTD-ILD include GGOs, fiber strips, sub-pleural interlobular septal thickening, small nodules, traction bronchiectasis, subpleural arc shadow, honeycomb lung changes (mainly concentrated in the middle and lower lungs), and cystic formations. Some specific imaging features are associated with an increased likelihood of progression and risk of death in interstitial lung abnormalities. For example, an increased extent of lung fibrosis on HRCT and definitive signs of fibrosis (e.g. pulmonary parenchymal architectural distortion) predict the highest risk of progression. Subpleural reticular marks suggest increased likelihood of progression as well as honeycombing and traction bronchiectasis, while centrilobular nodules may suggest a lower likelihood of progression. Both ‘probable UIP’ and ‘UIP patterns’ are related to increased risk of death (185). In addition, disease progression on imaging is associated with increasing age and *MUC5B* genotype copies (186, 187). Interestingly, computer-based computed tomography analysis (CALIPER) indicates that pulmonary vessel volume is an independent predictor of mortality in CTD-ILD patients (188).

Serum markers have also been investigated in the diagnosis and prognosis of ILD (189, 190). The presence of the same biomarkers suggests that CTD-ILD and IPF share a common pathophysiological process or mechanism (191, 192). In CTD-ILD, different biomarkers have been associated with worse outcome such as Krebs von den Lungen-6 (KL-6), cancer antigen 19-9 (CA 19-9), cancer antigen 125 (CA 125), vascular cell adhesion molecule-1 (VCAM-1), and C-X-C motif chemokine ligand 13 (CXCL13) (193). SSc-ILD prognostic biomarkers included, in addition to the above biomarkers, surfactant protein-D (SP-D), surfactant protein-A (SP-A), chitinase-3-like protein 1 (YKL-40), matrix metalloproteinases 12 (MMP12), tissue inhibitor of metalloproteinase-1 (TIMP-1), 16-kDa Clara cell secretory protein (CC16), (Tenascin C), C-C motif chemokine ligand 2 and 8 (CCL2 and CCL18), interleukins 6 and 2 (IL-6 and IL-2), C reactive protein (CRP), C-X-C motif chemokine ligand 4 and 10 (CXCL4 and CXCL10), and fractalkine (CX3CL1) (193).

Most importantly, rheumatologists must consider the clinical characteristics and radiographic findings of their patients and use serum biomarkers to aid their diagnostic work-up, and where appropriate, help in prognostication. KL-6 has the strongest value in diagnosing IPF and CTD-ILD, followed by SP-D, and MMPs as the most meaningful tools for IPF diagnosis. KL-6, SP-D, and chemokine ligand 18 (CCL18) have a high sensitivity but are not specific in diagnosing SSc-ILD, and CCL18 can predict the deterioration of IPF and SSc-ILD where CCL18 has a higher predictive value (194–208).

## Assessment and Treatment of CTD-ILD

The correct and timely diagnosis of CTD-ILD is necessary in order to delivery appropriate therapy. Once the diagnosis of CTD-ILD is established and extent of disease progression is assessed, then prognosis can be determined. An individualized treatment regime can then be initiated with regular clinic follow-ups (3, 209, 210). Indications to use GC and immunosuppressive agents depends on the primary disease, systemic activity, reversibility, and ILD clinical course. ILD can be divided into main IIP, rare IIP, and unclassified IIP (211). Main IIP can be further sub-divided into acute IIP (days to weeks), mainly AIP and OP; subacute IIP (weeks to months), mainly OP; and chronic IIP (months to years), mainly UIP and NSIP. The rare IIP is typically LIP. Patients with acute and subacute phase IIP need timely initiation of GC treatment combined with immunosuppressive therapy. For patients with chronic phase IIP such as honeycombed lung, high-dose GC and immunosuppressive therapy may not be beneficial. In this case, anti-pulmonary fibrosis treatment such as pirfenidone and nintedanib (212–214) may be considered.

The prognosis of CTD-ILD depends on ILD classification. The more urgent the course is, the better the effect of GC and immunosuppressive agents. On the other hand, the slower the course of the disease, as in NSIP and UIP, the poorer the efficacy of GC and immunosuppressive agents and the worse the prognosis. Considering the selection of immunosuppressive agents, there is currently no uniform management guideline for CTD-ILD. Rheumatologists should carefully consider the actual situation of each patient according to their underlying CTD, disease severity, the rate of disease progression (215–218) when selecting immunosuppressive agents [i.e. cyclophosphamide (CYC) (219–222), mycophenolic mofetil (223–225), azathioprine (226, 227), cyclosporine (228), tacrolimus (229, 230), and CD20 monoclonal antibody (231, 232)].

Several new therapeutic agents have been reported for the treatment of CTD-ILD, including Tripterygium wilfordii Hook F (233), tocilizumab, and abatacept. A clinical study reported the therapeutic efficacy of Tripterygium wilfordii Hook F as being comparable to CYC in the treatment of SSc-ILD when used only for maintenance therapy, but not for induction therapy (233). Biologics are also increasingly becoming available to treat ILD. Based on the rationale that elevated circulating IL-6 is predictive of progression in SSc-ILD (234), and the promising results from clinical trials (235–237), the FDA has approved tocilizumab in adult patients with SSc-ILD (218). Current evidence also indicates the promising efficacy and safety of abatacept in treating RA-ILD patients (238, 239).

In terms of anti-fibrosis therapy, the United States FDA has approved nintedanib, an inhibitor of multiple tyrosine kinases, for use in CTD-ILD with PF-ILD phenotype and SSc-ILD (218, 240). Two large randomized clinical trials (SENSCIS and INBUILD) showed that nintedanib reduced the annual rate of loss of FVC (212, 240). CTD-ILD with the PF-ILD phenotype and SSc-ILD patients who still exhibit disease progression after being treated with MMF or CYC may benefit from the addition of nintedanib to standard treatment (212, 240, 241). Although a pilot study has shown that administration of pirfenidone was associated with a reduction in dyspnea and an increase in vital capacity in SSc-ILD (242), other studies have not demonstrated a significant effect of pirfenidone (243), and therefore, the evidence for pirfenidone in these groups is less convincing (244). Other treatments include IVIG, plasmapheresis (245), and anti-reflux drugs. For end-stage or refractory cases, two promising novel therapeutic strategies such as autologous hematopoietic stem-cell transplantation and lung transplantation may be considered (246).

Clinical deterioration during routine follow-up should prompt the treating rheumatologists to consider the possible underlying causes: Acute exacerbation of CTD-ILD? Drug-induced ILD? Infection? Of note, when considering whether a patient’s presentation is due to acute exacerbation of CTD-ILD or infection, it is critically important to rule out infection before initiating immunosuppressive treatment (247–252). The exact cause of a patient’s exacerbation should be determined as expeditiously as possible. However, it is also possible that multiple concomitant factors are causing the exacerbation. Notably, it is important to recognize that infections and acute exacerbation of CTD-ILD can mimic one another, can coexist, and can promote each other (65, 248, 253–255).

A comprehensive clinical evaluation is required including: (a) evaluation of the patient’s occupation or living environment, (b) analysis of current or prior medication use, (c) systemic analysis of the patient’s symptoms, signs, imaging characteristics, and infection screening, and (d) evaluation of the patient’s immune function status (including neutrophils, humoral immunity, cellular immunity levels and functional status). The first step is to remove any suspicious drugs, then followed by initiation of specific therapy such as GC (as long as infection has been ruled out). If the diagnosis is still uncertain after this evaluation, for patients with mild disease, the diagnosis should be confirmed with more extensive or invasive examination. If the diagnosis remains elusive, then antimicrobial therapy may be considered first. If this approach is ineffective, then empiric treatment with a GC may be considered. For critically ill patients, treatment with combination antibiotics and a GC is generally recommended albeit clinical evidence is limited.

Supportive care including cessation of cigarette smoking, use of supplemental oxygen, annual influenza vaccination, and pneumococcal vaccination, should all be considered. In addition to treating the underlying disease, it is also necessary to treat comorbidities such as GERD, pulmonary hypertension, and sleep apnea. Consultation with a gastroenterologist, cardiologist, and pulmonologist is often necessary to formulate an appropriate treatment plan for the patient. Prognosis is related to the patient’s underlying disease, type of ILD, response to treatment, related comorbidities, as well as the patient’s education level and compliance with medical therapy (**Table 3**). The treating rheumatologist needs to actively educate patients, effectively communicate with patients and their families (including end-of-life considerations), and work with patients to develop individualized treatment plans.

## Future Directions

The clinical incidence of CTD-ILD is high. Each type of CTD-ILD has its distinct clinical characteristics, therapeutic response, and prognosis. In general, the lung is one of the important organs involved in CTD where symptoms can first arise and where lung function is an independent prognostic indicator in CTD. In light of the heterogeneity and complexity of CTD-ILD and its clinical manifestations and presentations, a multi-disciplinary collaborative effort with other clinical specialists is often necessary to further our understanding of CTD-ILD and to develop individualized treatment plans.

Our collective goal is to improve the early diagnosis and treatment of CTD-ILD in order to improve the prognosis and survival of patients. Looking into the near future, advanced research technologies using high-throughput genomics, proteomics, and metabolomics together with artificial intelligence will further pave the way and provide insight in identifying relevant mechanistic pathways and molecular targets for drug development and disease interventions.

## Author Contributions 

TS, XS, PL, and ZS wrote the main manuscript text and prepared all figures. XL and JS provided the image data. SY, WZ, SA, AZ, PL, and ZS jointly supervised this work. All authors contributed to the article and approved the submitted version.

## Funding

The Key Research and Development Projects of Anhui Province (1804h08020228).

## Conflict of Interest

The authors declare that the research was conducted in the absence of any commercial or financial relationships that could be construed as a potential conflict of interest.

## Glossary

AECAs: Anti-endothelial cell antibodies
anti-CCP: anti-cyclic citrullinated peptide
ARS: anti-aminoacyl-transfer RNA synthetase
ASS: anti-synthetase syndrome
BALF: bronchoalveolar lavage fluid
BECs: bronchial epithelial cells
CCL18: chemokine ligand 18
COP: cryptogenic organizing pneumonia
CTD: Connective tissue disease
CYC: cyclophosphamide
DAD: diffuse alveolar damage
DILD: drugs induced ILD
DIP: desquamative interstitial pneumonia
DLCO: diffusion capacity of the lung for carbon monoxide
DM: dermatomyositis
DMARDs: disease-modifying anti-rheumatic drugs
DPLDs: diffuse parenchymal lung diseases
EMT: epithelial-mesenchymal transition
FVC: forced vital capacity
GC: glucocorticoid
GERD: gastroesophageal reflux disease
GGOs: ground-glass opacities
*HDAC*: histone deacetylase
HRCT: high resolution computed topography
IIMs: idiopathic inflammatory myopathies
IIP: idiopathic interstitial pneumonia
ILD: interstitial lung disease
IPAF: interstitial pneumonia with autoimmune features
IPF: idiopathic pulmonary fibrosis
KL-6: Krebs von den Lungen-6
LIP: lymphoid interstitial pneumonia
LTL: leukocyte telomere length
MAAs: myositis-associated antibodies
MCTD: mixed connective tissue disease
MMPs: matrix metalloproteinases
MSAs: myositis-specific autoantibodies
MTX: methotrexate
NSIP: nonspecific interstitial pneumonia
OP: organizing pneumonia
PF-ILD: progressive fibrosing-ILD
PM: polymyositis
RA: rheumatoid arthritis
RB: respiratory bronchiolitis
RNP: ribonucleoprotein
RV: residual volume
SAFB: scaffold attachment factor B
SLE: systemic lupus erythematosus
SP-D: surfactant protein-D
SS: Sjogren’s syndrome
SSc: systemic sclerosis
TGF-β: transforming growth factor-β
TLC: total lung capacity
TLRs: Toll-like receptors
TREM-1: Triggering receptor expressed on myeloid cells type 1
UIP: usual interstitial pneumonia
*ZEB1*: zinc finger *E-box-homeobox 1*
